# Keeping carbon dioxide in check

**DOI:** 10.7554/eLife.27563

**Published:** 2017-05-17

**Authors:** Alfredo J Garcia, Jan-Marino Ramirez

**Affiliations:** 1 Institute for Integrative Physiology and the Section of Emergency Medicine, University of Chicago, Chicago, United States; 2 Center for Integrative Brain Research, Seattle Children's Research Institute, Seattle, United Statesjan.ramirez@seattlechildrens.org; 3 Department of Neurological Surgery, University of Washington School of Medicine, Seattle, United States

**Keywords:** arteriole, breathing, chemoreception, ATP, Rat

## Abstract

The response of the brainstem to increased levels of carbon dioxide in the blood is coordinated with the response of the cardiovascular system.

**Related research article** Hawkins VE, Takakura AC, Trinh A, Malheiros-Lima MR, Cleary CM, Wenker IC, Dubreuil T, Rodriguez EM, Nelson MT, Moreira TS, Mulkey DK. 2017. Purinergic regulation of vascular tone in the retrotrapezoid nucleus is specialized to support the drive to breathe. *eLife* **6**:e25232. doi: 10.7554/eLife.25232

Changes in the level of carbon dioxide molecules and hydrogen ions in the blood can change its pH, and this can have a negative impact on brain function. To avoid this, mammals rely on specialized cells in the brainstem called central chemoreceptors that can detect changes in the pH of the blood. When these chemoreceptors detect such a change, the body responds by regulating blood flow and breathing. However, changes in the rate at which blood flows through the brain make it more difficult to detect changes in its pH.

The pH of a liquid is determined by the concentration of hydrogen ions in it: the higher the concentration of hydrogen ions, the lower the pH. Carbon dioxide influences the pH of blood by reacting with water to form carbonic acid (H_2_CO_3_), which can dissociate to form a hydrogen ion (H^+^) and a hydrogen carbonate ion (HCO_3_^-^). Increasing the concentration of carbon dioxide in the blood therefore results in more H^+^ ions and a lower pH. However, both these reactions are reversible, and breathing heavily to remove carbon dioxide from the body will lead to a reduction in the concentration of the H^+^ and HCO_3_^-^ ions, and hence to an increase in pH.

For over a century, it was thought that all the blood vessels in the brain reacted to increased levels of carbon dioxide in the blood by becoming wider to increase blood flow. Now, in eLife, Daniel Mulkey of the University of Connecticut, Thiago Moreira of the University of Sao Paulo and colleagues – including Virginia Hawkins as first author – report that elevated levels of carbon dioxide (a condition known as hypercapnia) cause the blood vessels in the brainstem to become narrower, while the blood vessels in the rest of the brain become wider (Hawkins et al., 2017).

Although the magnitude of the narrowing observed in the brainstem is modest (the diameter of the arteriole is reduced by less than 10%), the phenomenon reported by Hawkins et al. is reminiscent of the way that a shortage of oxygen (a condition known as hypoxia) causes the small pulmonary arteries in the lung to become narrower. This process optimizes lung function by redirecting of blood flow to areas of the lung where there is little blood flow, thereby increasing the surface area for gas exchange (Ward and McMurtry, 2009). Similarly, the narrowing of the blood vessels in the brainstem caused by increased levels of carbon dioxide might, according to Hawkins et al., help the body to measure the levels of carbon dioxide and H^+^ ions in the blood more accurately.

While neurons throughout the brainstem are known to be involved in the detection of carbon dioxide and H^+^ ions (Guyenet et al., 2010), the neurons in two regions of the brainstem – the ventrolateral medulla and the retrotrapezoid nucleus – have a particularly significant role (Kumar et al., 2015). However, the discovery in 2010 that astrocytes (cells in the brain and spinal cord that are not neurons) were also involved in central chemoreception showed that the regulation of breathing was more complex than expected (Gourine et al., 2010). The results of the elegant study by Hawkins et al. are further evidence in support of such complexity.

These are exciting times for the field. For over a half of a century, the drive to understand central chemosensitivity has understandably been focused on the cellular and molecular substrates of the phenomenon. However, growing evidence supports the notion that central chemosensitivity is a property that emerges from concerted interactions across the multiple cell types in the neurovascular unit, and that physiological interactions have an important role. While the phenomenon reported by Hawkins et al. appears to be small in magnitude, its potential impact on physiology cannot be dismissed.

Further research is now needed to address a number of questions: Does the constriction of the blood vessels seen by Hawkins et al. influence the pH of the surrounding tissue? Does the constriction have an impact on the cellular physiology of the neurons and astrocytes in the neurovascular unit? And how do the blood vessels in other regions of the brainstem respond to high levels of carbon dioxide? Answering these questions could, ultimately, lead to a systems-level understanding of the mechanisms underlying central chemosensitivity, and thus provide insights into the variability of this process in both health and disease.
